# Role of KRAS in regulating normal human airway basal cell differentiation

**DOI:** 10.1186/s12931-019-1129-4

**Published:** 2019-08-09

**Authors:** Fumihiro Ogawa, Matthew S. Walters, Afrah Shafquat, Sarah L. O’Beirne, Robert J. Kaner, Jason G. Mezey, Haijun Zhang, Philip L. Leopold, Ronald G. Crystal

**Affiliations:** 1 Department of Genetic Medicine, Weill Cornell Medical College, 1300 York Avenue, Box 164, New York, NY 10065 USA; 2 Pulmonary, Critical Care & Sleep Medicine, Department of Medicine, University of Oklahoma Health Sciences Center, Oklahoma City, OK USA; 3 Computational Biology, Cornell University, Ithaca, NY USA

**Keywords:** Stem/progenitor, Airway, Basal cell, KRAS, Differentiation, Cigarette smoking

## Abstract

**Background:**

KRAS is a GTPase that activates pathways involved in cell growth, differentiation and survival. In normal cells, KRAS-activity is tightly controlled, but with specific mutations, the KRAS protein is persistently activated, giving cells a growth advantage resulting in cancer. While a great deal of attention has been focused on the role of mutated KRAS as a common driver mutation for lung adenocarcinoma, little is known about the role of KRAS in regulating normal human airway differentiation.

**Methods:**

To assess the role of KRAS signaling in regulating differentiation of the human airway epithelium, primary human airway basal stem/progenitor cells (BC) from nonsmokers were cultured on air-liquid interface (ALI) cultures to mimic the airway epithelium in vitro. Modulation of KRAS signaling was achieved using siRNA-mediated knockdown of KRAS or lentivirus-mediated over-expression of wild-type KRAS or the constitutively active G12 V mutant. The impact on differentiation was quantified using TaqMan quantitative PCR, immunofluorescent and immunohistochemical staining analysis for cell type specific markers. Finally, the impact of cigarette smoke exposure on KRAS and RAS protein family activity in the airway epithelium was assessed in vitro and in vivo.

**Results:**

siRNA-mediated knockdown of KRAS decreased differentiation of BC into secretory and ciliated cells with a corresponding shift toward squamous cell differentiation. Conversely, activation of KRAS signaling via lentivirus mediated over-expression of the constitutively active G12 V KRAS mutant had the opposite effect, resulting in increased secretory and ciliated cell differentiation and decreased squamous cell differentiation. Exposure of BC to cigarette smoke extract increased KRAS and RAS protein family activation in vitro. Consistent with these observations, airway epithelium brushed from healthy smokers had elevated RAS activation compared to nonsmokers.

**Conclusions:**

Together, these data suggest that KRAS-dependent signaling plays an important role in regulating the balance of secretory, ciliated and squamous cell differentiation of the human airway epithelium and that cigarette smoking-induced airway epithelial remodeling is mediated in part by abnormal activation of KRAS-dependent signaling mechanisms.

**Electronic supplementary material:**

The online version of this article (10.1186/s12931-019-1129-4) contains supplementary material, which is available to authorized users.

## Background

The RAS protein family are a class of small GTP-binding proteins that function as signal transduction molecules to regulate many cellular processes including proliferation, differentiation, and apoptosis [1–4]. The proto-oncogene KRAS, one of three human Ras genes, has intrinsic GTPase activity which catalyzes the hydrolysis of bound GTP to GDP which inactivates the signal [2, 5]. Specific mutations in KRAS lock the protein into the GTP-bound state resulting in constitutive signaling which gives mutated cells a growth advantage and leads to development of cancer [5–8]. KRAS mutations play a major role in the pathogenesis of lung adenocarcinoma, representing > 25% of the driver mutations for lung adenocarcinomas [8–11]. While a great deal of attention is focused on the role of KRAS in the pathogenesis of lung cancer, little is known about the role of KRAS in regulating normal human airway epithelial function, nor of the direct impact of cigarette smoking, the major cause of lung cancer, on KRAS activation.

Based on the knowledge that KRAS signaling regulates a diverse array of cellular pathways relevant to differentiation [2, 12–15], we hypothesized that, independent of the role of mutant KRAS as a driver mutation for lung cancer, modulation of KRAS expression and/or activity in the normal airway epithelium has a significant influence on normal airway epithelial differentiation and that cigarette smoking, a stress associated with abnormal epithelial differentiation, dysregulates airway epithelial differentiation in part by abnormal activation of KRAS. To assess this hypothesis, we capitalized on the knowledge that in the human airway epithelium, basal cells (BC) function as stem/progenitor cells and differentiate into ciliated and secretory cells during normal turnover and in response to environmental insult [16–30]. To assess the role of KRAS signaling in regulating differentiation of the human airway epithelium, primary human airway BC cultured on air-liquid interface (ALI) cultures were used as a model to mimic the airway epithelium in vitro. The results demonstrate that siRNA-mediated knockdown of KRAS has a significant impact on BC differentiation resulting in decreased differentiation into secretory and ciliated cells with a corresponding shift toward squamous cell differentiation. Conversely, constitutive KRAS signaling via lentivirus mediated over-expression of constitutively active KRAS has the opposite effect resulting in increased secretory and ciliated cell differentiation and decreased squamous cell differentiation. Relevant to smoking, the data demonstrates that cigarette smoke extract (CSE) treatment of BC under non-differentiating and differentiating ALI culture conditions increases KRAS and RAS protein family activation in vitro and that in vivo airway epithelial brushings from healthy smokers display elevated RAS activation compared to nonsmokers. Together, these data demonstrate that KRAS-dependent signaling plays an important role in regulating differentiation of the human airway epithelium, and suggest that the development of airway epithelial remodeling in smokers may, in part, be regulated by cigarette smoke-mediated activation of KRAS-dependent signaling in BC.

## Methods

### Culture of primary human airway basal cells

Nonsmoker primary basal cells (BC) were purchased from Lonza (CC2540S, Walkersville, MD). All cultures were seeded at 3000 cells/cm^2^ into T75 flasks and maintained in Bronchial Epithelial Growth Media (BEGM, Lonza) before differentiation on air-liquid interface (ALI) as described [31–34]. To investigate the differentiation of BC into a mucociliated epithelium, the BC were grown on air-liquid interface (ALI) culture. Briefly, BC were trypsinized and seeded at a density of 3.75 × 10^5^ cells/cm^2^ onto 0.4 μm pore-sized Transwell inserts (Corning, Corning, NY) pre-coated with human type IV collagen (Sigma, St Louis, MO). The initial culture medium consisted of a 1:1 mixture of DMEM (Cellgro, Manassas, VA) and Ham’s F-12 Nutrient Mix (GIBCO-Invitrogen, Carlsbad, CA) containing 5% fetal bovine serum, 1% penicillin-streptomycin, 0.1% gentamycin and 0.5% amphotericin B. The following day, the medium was changed to 1:1 DMEM/Ham’s F12 (including antibiotics described above) with 2% Ultroser G serum substitute (BioSerpa S.A., Cergy-Saint-Christophe, France). Once the cells had reached confluence 2 days post seeding, the media was removed from the upper chamber to expose the apical surface to air and establish the ALI (referred to as ALI Day 0). The ALI cultures were then grown at 37 °C, 8% CO_2_, with fresh media changed every 2 to 3 days. Following 5 days on ALI, the CO_2_ levels were reduced to 5% until harvest of the cultures at the desired time point. For histological analysis ALI trans-well inserts were fixed for paraffin embedding and sectioning (performed by Histoserv, Germantown, MD). For general histology, sections were stained using standard protocols for hematoxylin and eosin (H&E) or Alcian blue.

### siRNA-mediated knockdown of KRAS

Basal cells were used without transfection or were transfected with 5 pmol of control siRNA (AM4635, Thermo Scientific, Waltham, MA) or KRAS specific siRNA (AM51331, Thermo Scientific) using Lipofectamine RNAiMAX Reagent and Opti-MEM media (both from Life Technologies) at the time of seeding cells for ALI culture [33]. The next day, the standard protocol for ALI culture was continued.

### Lentivirus-mediated over-expression of KRAS

The cDNA sequence of human wild type (WT; 46746, Addgene, Cambridge, MA) and the constitutively active mutant G12 V mutant of KRAS (52729, Addgene) were PCR amplified using specific primers (forward: 5′-GGTGGAGTATTTGATAGTGTATTAACC-3′; reverse: 5′-AGAATGGTCCTGCACCAGTAA-3′) and cloned into the TOPO® TA subcloning vector (Invitrogen) using the manufacturer’s protocol. The KRAS inserts were then subcloned into the multiple cloning site of pCDH-MSCV-MCS-EF1α-GFP lentiviral vector (CD711B-1, System Biosciences, Mountain View, CA) via the Nhe I and BamHI restriction sites. The resulting plasmids were sequenced to verify the integrity of the KRAS open reading frame. Recombinant replication deficient lentiviruses were generated by transient co-transfection of 293A cells with the KRAS lentiviral vectors and the appropriate packaging plasmids pGal-Pol and pMD.G (VSVg envelope) as previously described [32]. Virus titer was measured using the Lenti-X™ qRT-PCR Titration Kit (631235, Clontech Laboratories, Mountain View, CA). For all experiments, BC were infected at a multiplicity of infection (MOI) of 50 [32].

### Quantitative PCR

Total RNA was extracted using TRIzol (Invitrogen) and purified using the Rneasy MinElute RNA purification kit (Qiagen, Valencia, CA). Double-stranded cDNA was synthesized from 1 μg of total RNA using TaqMan Reverse Transcription Reagents (Applied Biosystems, Foster City, CA). Gene expression was assessed using TaqMan quantitative PCR and relative expression levels determined using the ΔCt method with 18S ribosomal RNA as the endogenous control [31–34]. Premade TaqMan Gene Expression Assays were obtained from Applied Biosystems: KRAS (Hs00364284_g1); KRT5 (Hs00361185_m1); TP63 (Hs00978343_m1); MUC5AC (Hs01365616_m1); MUC5B (Hs00861588_m1); SCGB1A1 (SCGB1A1, Hs00171092_m1); DNAI1 (Hs00201755_m1); FOXJ1 (Hs00230964_m1); KRT6B (Hs00745492_s1) and IVL (Hs00846307_s1). Additional premade TaqMan Gene Expression probes were obtained from ThermoFisher Scientific: MKI67 (Hs01032443_m1); SOX2 (Hs01053049_s1); SOX9 (Hs00165814_m1); NOTCH1 (Hs01062014_m1); NOTCH2 (Hs01050702_m1); NOTCH3 (Hs01128537_m1); HES1 (Hs00172878_m1); HEY1 (Hs05047713_s1); and HEY2 (Hs01012057_m1).

### Western analysis

Western analysis was performed as described [31–34]. Briefly, cells were harvested and lysed in radioimmunoprecipitation lysis (RIPA) buffer (Sigma) containing complete protease inhibitor cocktail (Roche, Mannheim, Germany) and Halt^TM^ phosphatase inhibitor cocktail (Pierce, Rockford, IL). The protein concentration was then quantified using the Bradford Assay and an appropriate volume of 4X NuPAGE LDS sample buffer (Invitrogen) containing 200 mM dithiothreitol (DTT) added to each sample. The cellular lysates were then boiled for 5 min and equal amounts of total protein for each sample analyzed using NuPAGE 4–12% Bis-Tris gradient gels (Invitrogen) and subsequently transferred onto nitrocellulose membranes with a Bio-Rad semidry apparatus before Western analysis. The primary antibodies used were KRAS (1/4000; 3965S; Cell Signaling Technology, Danvers, MA) and GAPDH (1/5000, SC-32233; Santa Cruz Biotechnology, Santa Cruz, CA).

### Immuno-staining

Immunohistochemical and immunofluorescent staining was performed on normal human bronchus tissue (HuFPT111, US Biomax, Rockville, MD) or ALI cross-sections as described [31–34]. The following primary antibodies were used and incubated at 4 °C overnight for each staining: rabbit polyclonal KRAS (10 μg/ml; NBP1–58261; Novus Biologicals); rabbit polyclonal KRT5 (2 μg/ml; PA1–37974; ThermoFisher Scientific); rabbit polyclonal MUC5B (4 μg/ml; sc-20119; Santa Cruz Biotechnology); SCGB1A1 (5 μg/ml; RD181022220; BioVendor LLC, Candler, NC); mouse monoclonal β-tubulin IV (10 μg/ml; MU178-UC; Biogenex, Fremont, CA) and mouse monoclonal IVL (2 μg/ml; MS-126-P; Thermo Scientific). Isotype matched IgG (Jackson Immunoresearch Laboratories, West Grove, PA) was the negative control. The Vectastain Elite ABC kit and AEC substrate kit (Dako North America, Carpinteria, CA) were used to visualize antibody binding for immunohistochemistry and the slides were counterstained with Mayer’s hematoxylin (Polysciences, Warrington, PA) and mounted using faramount mounting medium (Dako North America). To visualize the antibody binding for immunofluorescence, Alexa Fluor® 488 Goat Anti-Mouse IgG (A-11029), Alexa Fluor® 546 Goat Anti-Rabbit IgG (A-11035), Alexa Fluor® 488 Donkey Anti-Goat IgG (A-11055) and Alexa Fluor® 555 Donkey Anti- Rabbit IgG (A-31572; all from Life Technologies) labeled secondary antibodies were used. The cells were counterstained with DAPI to identify cell nuclei and subsequently mounted using ProLong® Gold antifade reagent (Invitrogen).

### Quantification of epithelial thickness and differentiation

Epithelial thickness of ALI cultures was quantified on H&E stained cross-sections. For each cross-section, 20 images equally distributed between both ends of the sectioned membrane were acquired and three measurements were made at one quarter, one half, and three-quarter intervals with Image J software (https://imagej.nih.gov/ij/, Version1.45 s, National Institute of Health, Bethesda, MD). For quantification of BC differentiation at the histological level via immuno-staining using cell type specific markers, a minimum of 15 images equally distributed between both ends of the sectioned membrane were acquired and a minimum of 500 total cells counted for each individual experiment. 

### Generation of cigarette smoke extract

Cigarette smoke extract (CSE) was made from the smoke of one Marlboro Red commercial cigarette bubbled through 12.5 ml of differentiation medium that was then filtered through a 0.2 μm pore filter as described [31]. To ensure standardization between experiments and batches of CSE, the absorbance was measured at 320 nm on a spectrophotometer and the optical density of 1 was defined as 100%. The CSE was frozen in single use aliquots at − 20 °C and then applied to cells at each media change to the desired concentration.

### Quantification of KRAS and RAS protein family activation

Levels of activated KRAS were quantified using the KRAS activation Kit (ab211159; Abcam) according to the manufacturers’ protocol. This kit uses GST-Raf1-RBD (Rho binding domain) fusion protein to bind the activated form of GTP-bound Ras, which can then be co-immunoprecipitated with glutathione resin from cell lysates. For each sample, 500 μg of total protein lysate were used per assay. Levels of total (input) and activated (elution following co-immunoprecipitation) KRAS are determined by Western analysis using a rabbit polyclonal KRAS antibody. Levels of activated KRAS were quantified using ImageJ software with the untreated cells set as the reference for 100% activity.

To quantify the effect of CSE exposure on RAS protein family activity, the 96-well RAS Activation ELISA Kit (STA-440; Cell Biolabs, San Diego, CA) was used according to the manufacturers’ protocol. The kit uses the Raf1 RBD (Rho binding domain) attached to a 96-well plate to selectively pull down the active form of RAS (GTP bound) from cell lysates. The captured GTP-RAS is then detected by a pan-RAS antibody and HRP-conjugated secondary antibody, with the absorbance read on a spectrophotometer at a wavelength of 450 nm. For each sample, 10 μg of total protein lysate were used per well. Levels of activated RAS in the untreated and nonsmoker cells were set as the reference for 100% activity.

### Study population and sampling of the airway epithelium

Healthy nonsmokers (*n* = 5) and smokers (*n* = 5) were recruited under IRB-approved protocols (Table 1). Following consent (see Additional file 1 for full inclusion/exclusion criteria) fiberoptic bronchoscopy was utilized to collect large airway epithelial cells from each subject [17]. Briefly, the airway epithelial cells were obtained by gently gliding the brush back and forth on the airway epithelium 5–10 times in up to 10 different locations in the same general area. Cells were detached from the brush by flicking into 5 ml of ice-cold Bronchial Epithelium Basal Medium (BEGM, Lonza, Basel, Switzerland). An aliquot of 0.5 ml was reserved for protein extraction.

**Table 1:** Demographics of Nonsmokers and Smokers^a^

Parameter	Nonsmokers	Smokers	Smokers vs nonsmokers *p* value
n	5	5	
Gender (M/F)	1/4	4/1	0.06
Age (yr)	33.4 ± 11.5	48.6 ± 8.1	0.05
Race (B/W/H/O)^b^	2/1/0/2	2/0/2/1	0.34
Body mass index	23.6 ± 2.0	23.4 ± 4.0	0.9
Smoking history			
Age of initiation	NA	20.4 ± 4.8	NA
Duration of smoking (pk-yr)	NA	25.1 ± 15.3	NA
Urine nicotine (ng/ml)^c^	< 2	640 ± 746	0.07
Urine cotinine (ng/ml)^c^	< 5	925 ± 312	0.002
Carboxyhemoglobin (%)	1.6 ± 0.2	2.6 ± 0.33	0.0008
Pulmonary function parameters^d^			
FVC	100.4 ± 17.4	114 ± 15.3	0.23
FEV1	101.2 ± 12.6	115 ± 17.2	0.19
% Change FEV1 post-bronchodilator	3.2 ± 3.6	3.4 ± 2.2	0.92
FEV1/FVC	85.4 ± 5.0	81.6 ± 3.8	0.22
TLC	98.6 ± 15.3	98.8 ± 6.7	0.98
DLCO	86.6 ± 7.6	88.2 ± 8.4	0.76
Cough Score^e^	0.6 ± 0.5	1 ± 1	0.46
Sputum Score^e^	0.4 ± 0.5	1 ± 1	0.28

^a^Data are presented as mean ± standard deviation, *p* values of numeric parameters calculated using a 2-tailed Student’s t-test with unequal variance, *p* value of categorical parameters calculated using a chi-square test for screening date

^b^Abbreviations^:^ B=Black, W=White, H=Hispanic, O=Other, NA = not applicable

^c^Undetectable urine nicotine < 2 ng/ml; cotinine < 5 ng/ml

^d^Pulmonary function testing parameters are given as % of predicted value with the exception of FEV1/FVC, which is reported as % observed; FVC - forced vital capacity, FEV1 - forced expiratory volume in 1 s, TLC -total lung capacity, DLCO - diffusing capacity

^e^Cough and sputum score were each evaluated on a scale of 0–4: 0 = not at all; 1 = only with chest infections; 2 = a few days a month; 3 = several days per week; 4 = most days per wk. [35]

### Statistical analysis

All data is presented as the mean ± standard error. Comparisons between two conditions were performed using an unpaired, two tailed Student’s t test for unequal variance. For experiments requiring multiple comparisons, ANOVA was performed with Tukey’s test to estimate the statistical significance for the contrasts across different groups. For experiments requiring multiple comparisons with time as a factor, a repeated measures ANOVA was performed with Tukey’s test to estimate the statistical significance for the contrasts across different groups.

## Results

### KRAS expression in the normal airway epithelium in vivo and in vitro

**Fig. 1:**
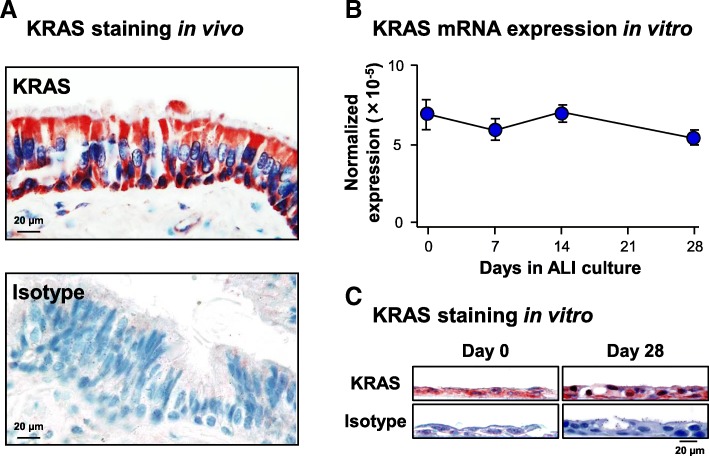
KRAS expression in the normal human airway epithelium in vivo and in vitro **a** Immunohistochemical staining analysis of KRAS in the airway epithelium. Normal nonsmoker human airway sections were analyzed for expression of KRAS. Isotype specific antibody was used as negative control. Scale bar 20 μm. **b** Expression of KRAS during differentiation of human airway basal cells (BC) on air-liquid interface (ALI) culture. KRAS mRNA expression was assessed by qPCR. Data points indicate the mean of *n* = 3 independent experiments, each performed in triplicate with independent donors of BC. Error bars indicate standard error of the mean. **c** Immunohistochemical staining analysis of KRAS during ALI culture. ALI day 0 and day 28 sections were analyzed for expression of KRAS. Isotype specific antibody was used as negative control. Scale bar 20 μm

To investigate KRAS expression and cellular localization in the human airway epithelium in vivo, KRAS immunohistochemistry staining was carried out with sections of normal human bronchus. The staining demonstrated KRAS is expressed ubiquitously in all cell types of the airway epithelium (Fig. 1a). Consistent with the in vivo data, mRNA expression and protein localization of KRAS in vitro demonstrated similar findings. Nonsmoker derived primary human airway basal cells (BC) were differentiated on ALI culture for 28 days into a mucociliated epithelium and harvested at multiple time points for analysis. At the mRNA level, KRAS expression remained constant throughout the differentiation process and no significant difference in expression was observed between ALI day 0 and day 28 of the differentiation process (Fig. 1b). These data were verified at the protein level via immunohistochemistry staining of ALI day 0 and day 28 sections which demonstrated ubiquitous staining of KRAS throughout the whole airway epithelium at each time point (Fig. 1c).

### Suppression of KRAS expression decreases secretory and ciliated cell differentiation and increases squamous cell differentiation

**Fig. 2:**
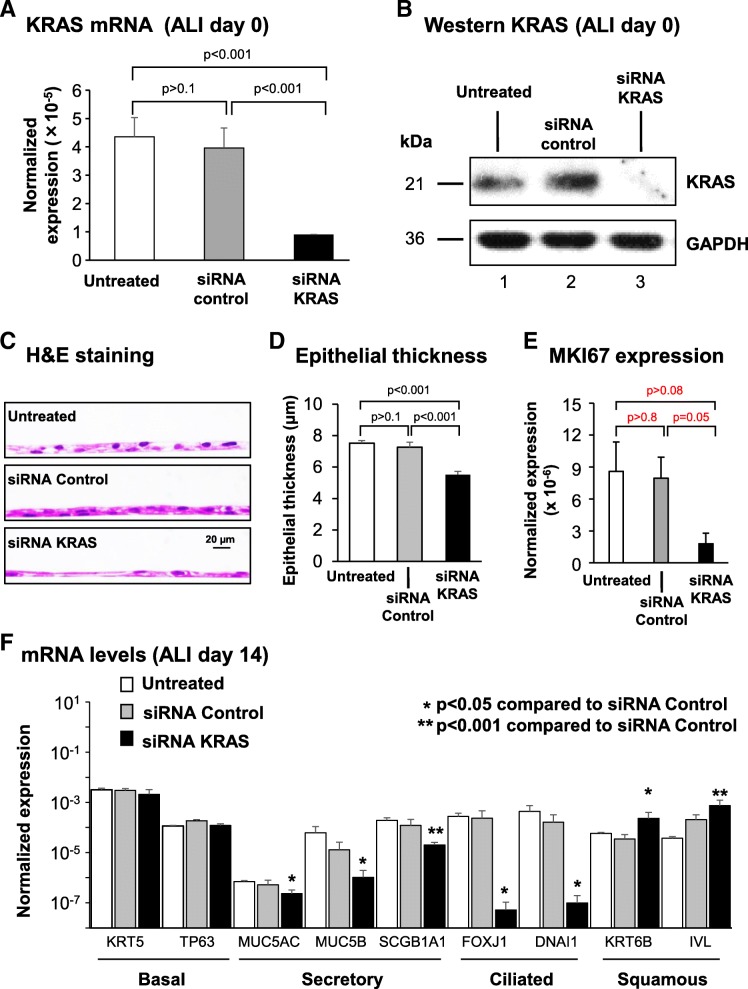
Effect of silencing of KRAS expression on regulation of basal cell (BC) differentiation into a mucociliated epithelium. Primary human airway BC were untreated or transfected with either control, or KRAS specific siRNA and cultured on ALI for 14 days to assess the impact of KRAS on BC differentiation. **a** qPCR analysis to assess mRNA expression of KRAS to confirm efficacy of siRNA-mediated knockdown at ALI day 0. Bars indicate the normalized gene expression. Error bars indicate standard error of the mean. Data from *n* = 3 independent experiments, each performed in triplicate with independent donors of BC. **b** Western analysis of KRAS expression following siRNA mediated knockdown at ALI day 0. Lane 1- untreated; lane 2- siRNA control; and lane 3- siRNA KRAS. GAPDH was used as a loading control. **c** Histology of untreated, siRNA control and siRNA KRAS cells at ALI day 14. **d** Quantification of epithelial thickness of ALI day 14. Bars indicate the epithelial thickness. Error bars indicate standard error of the mean. Data from *n* = 3 independent experiments, each performed with an independent donor of BC. **e** qPCR analysis to assess mRNA expression of the proliferation marker MKI67 at ALI day 14. Bars indicate the normalized mRNA expression. Error bars indicate standard error. Data from *n* = 3 independent experiments, each performed in triplicate with independent donors of BC. **f** qPCR analysis to assess mRNA expression of BC markers (KRT5, TP63), secretory cell markers (MUC5AC, MUC5B, SCGB1A1), ciliated cell markers (FOXJ1, DNAI1) and squamous cell markers (KRT6B, IVL) at ALI day 14. Bars indicate the normalized mRNA expression. Error bars indicate standard error. Asterisks indicate *p* < 0.05 (*) or *p* < 0.001 (**). ANOVA was used to determine the statistical significance among groups as described in the methods section. Data from *n* = 3 independent experiments, each performed in triplicate with independent donors of BC

To assess the role of KRAS signaling in regulating differentiation of the human airway epithelium, primary BC cultured on ALI were used as a model to mimic the airway epithelium in vitro. Suppression of KRAS-signaling was performed via siRNA mediated knockdown of KRAS expression. As controls, cells were either untreated or treated with scrambled siRNA. Cells with knockdown of KRAS consistently produced a leaky epithelium that failed to regenerate a fully differentiated mucocilated epithelium and survive to ALI day 28 (not shown). Therefore, all the analyses to characterize the effect of KRAS knockdown were performed at ALI day 14 when the epithelium was still viable. Treatment of cells with control siRNA had no significant effect on the expression of KRAS mRNA and protein compared to untreated cells (Fig. 2a, b). However, treatment of cells with KRAS specific siRNA resulted in a significant decrease (− 4.4-fold, *p* < 0.001) in KRAS mRNA levels at ALI day 0 relative to control siRNA (Fig. 2a). This was further validated at the protein level by Western analysis (Fig. 2b).

**Fig. 3:**
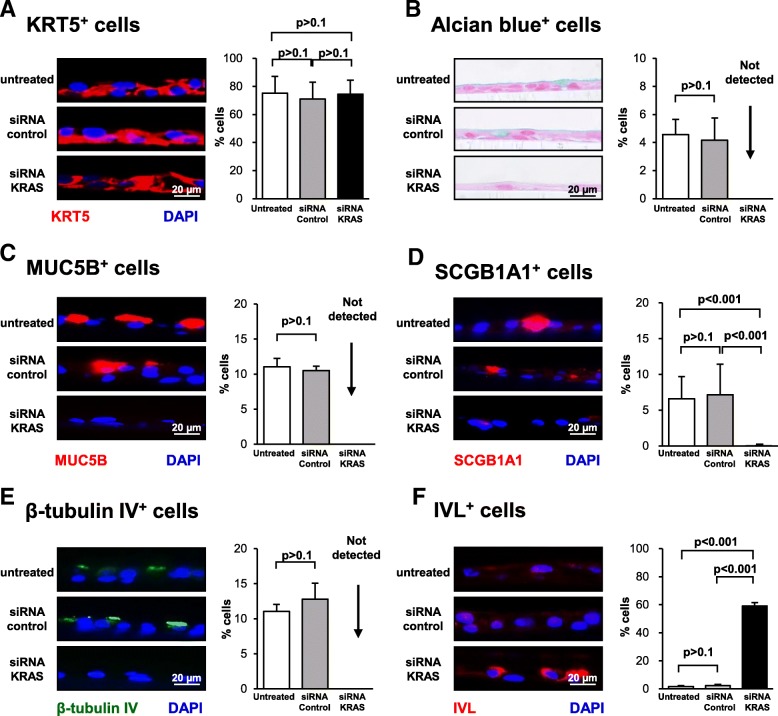
Effect of silencing KRAS expression on regulation of basal cell (BC) differentiation into a mucociliated epithelium. Primary human airway BC were untreated or transfected with either control, or KRAS specific siRNA and cultured on ALI for 14 days to assess the impact of KRAS on BC differentiation into a mucociliated epithelium. **a** Immunofluorescence staining of KRT5^+^ BC. Sections of cells on ALI day 14 stained for KRT5 (red) and DAPI (nuclei, blue). **b** Alcian blue staining of secretory cells. Sections of cells on ALI day 14 stained with Alcian blue (blue). **c** Immunofluorescence staining of MUC5B^+^ secretory cells. Sections of cells on ALI day 14 stained for MUC5B (red) and DAPI (nuclei, blue). **d** Immunofluorescence staining of SCGB1A1^+^ secretory cells. Sections of cells on ALI day 14 stained for SCGB1A1 (red) and DAPI (nuclei, blue). **e** Immunofluorescence staining of β-tubulin IV^+^ ciliated cells. Sections of cells on ALI day 14 stained for β-tubulin IV (green) and DAPI (nuclei, blue). **f** Immunofluorescence staining of IVL^+^ squamous cells. Sections of cells on ALI day 14 stained for IVL (red). The data for **a**-**f** are the mean percentage of positively stained cells for *n* = 3 independent experiments performed with independent donors of BC. Error error bars indicate standard error of the mean. ANOVA was used to determine the statistical significance among groups as described in the methods section. Scale bar 20 μm

Histological analysis of ALI day 14 cross-sections with H&E staining demonstrated that both untreated and siRNA control treated cells generated a mucociliated epithelium of comparable thickness (Fig. 2c, d). In contrast, silencing of KRAS expression during differentiation resulted in the appearance of a significantly thinner epithelium compared to siRNA control-treated cells (− 1.3-fold, *p* < 0.001). In support of these findings, qPCR analysis of the proliferation marker MKI67 demonstrated no significant (*p* > 0.8) difference in expression between untreated and siRNA control treated cells. However, compared to control siRNA treated cells knockdown of KRAS decreased expression of MKI67 (− 4.4 fold) suggesting suppression of KRAS decreases proliferation (Fig. 2e). However, despite a clear trend in expression differences between conditions the results were not significant (*p* = 0.05). To further characterize these differences, ALI day 14 cultures from each group were analyzed by qPCR for expression of cell type specific markers relevant to mucociliated [17, 20, 22, 34] and squamous differentiation [36, 37]. Consistent with the histology, comparison of untreated and siRNA control-treated cells demonstrated no significant differences in expression of BC (KRT5 and TP63), secretory (MUC5AC, MUC5B and SCGB1A1), ciliated (FOXJ1 and DNAI1) and squamous (KRT6B and IVL) markers (Fig. 2f). Compared to siRNA control-treated cells, no significant difference in expression of BC markers was observed following silencing of KRAS expression (Fig. 2f). However, KRAS silencing significantly decreased expression of secretory cell markers (− 2.2-fold MUC5AC, − 12.6-fold MUC5B, and − 5.9-fold SCGB1A1; all *p* < 0.05) and suppressed expression of ciliated cell markers (FOXJ1 and DNAI1; Fig. 2f). In contrast, a significant increase in expression of squamous cell markers (3.6-fold KRT6B and 6.6-fold IVL) was observed (Fig. 2f). These differentiation changes observed in the mRNA expression data were validated at the histological level by staining of ALI day 14 sections. Immunofluorescent staining of the BC marker KRT5 demonstrated no significant difference in positive cells between untreated, siRNA control and KRAS siRNA treated cells (75.1% untreated vs 71.0% siRNA control and 74.5% siRNA KRAS; Fig. 3a). Silencing of KRAS resulted in depletion of Alcian blue positive cells (4.6% untreated vs 4.2% siRNA control vs not detected siRNA KRAS-treated cells), MUC5B positive secretory cells (11.0% untreated vs 10.5% siRNA control vs not detected in siRNA KRAS-treated cultures), and a significant (*p* < 0.001) decrease in the number of SCGB1A1 (6.6% untreated vs 7.2% siRNA control and 0.02% siRNA KRAS) compared to siRNA control treated cells (Fig. 3b-d). In addition, no β-tubulin IV positive ciliated cells were observed following knockdown of KRAS (11.0% untreated vs 12.8% siRNA control vs not detected in siRNA KRAS-treated cells) (Fig. 3e). Conversely, a significant increase (all p < 0.001) in the number of IVL positive squamous cells (1.6% untreated vs 2.3% siRNA control vs 59.1% siRNA KRAS-treated cells) was observed following suppression of KRAS expression (Fig. 3f). These data suggest that KRAS expression is critical for regulating differentiation of BC into a mucociliated epithelium, with suppression of KRAS expression resulting in a diversion of differentiation toward the squamous cell lineage at the expense of secretory and ciliated cell differentiation.

### Over-expression of activated KRAS increases secretory and ciliated cell differentiation and decreases squamous cell differentiation

**Fig. 4:**
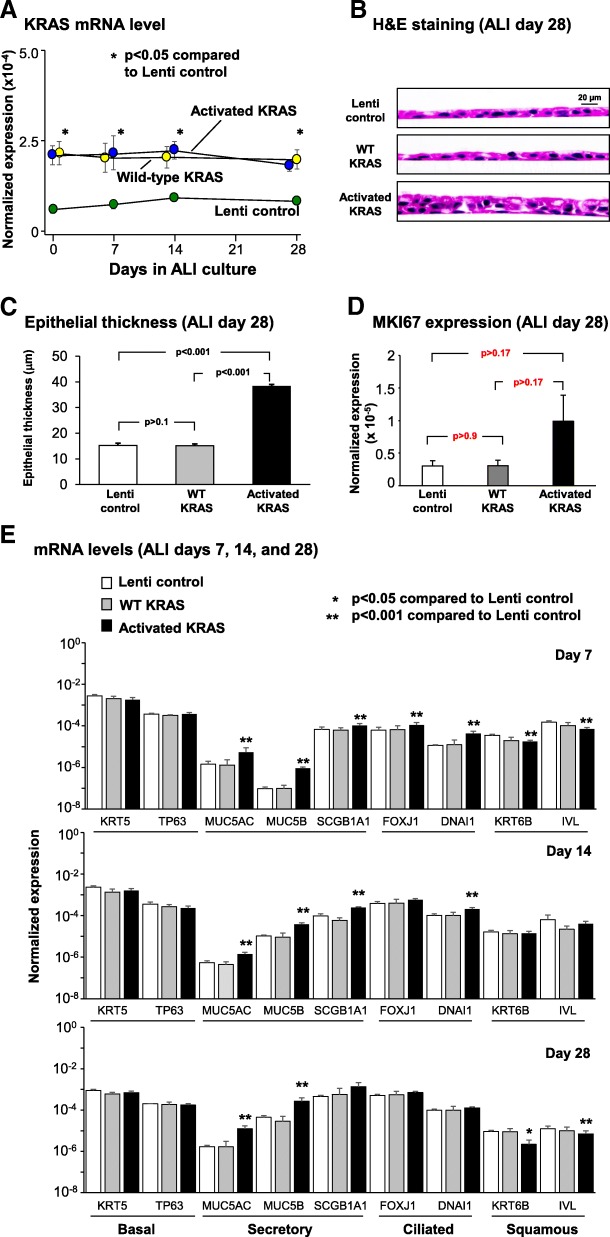
Effect of constitutive KRAS activity on promoting basal cell (BC) differentiation into secretory and ciliated cells. Primary human airway BC were infected with control lentivirus or lentivirus over-expressing wild-type (WT) KRAS or the constitutively active G12 V mutant (activated) and cultured on ALI for 28 days to assess the impact of KRAS on BC differentiation into a mucociliated epithelium. **a** qPCR analysis to assess mRNA expression of KRAS to confirm over-expression of KRAS during ALI culture. Data points indicate the mean expression and error bars indicate standard error of the mean. Data from *n* = 3 independent experiments, each performed in triplicate with independent donors of BC. **b** Histology of Lenti control, WT KRAS and activated KRAS cells at ALI day 28. **c** Quantification of epithelial thickness of ALI day 28. Bars indicate epithelial thickness. Error bars indicate standard error. Data from *n* = 3 independent experiments, each performed with an independent donor of BC. **d** qPCR analysis to assess mRNA expression of the proliferation marker MKI67 at ALI day 28. Bars indicate the normalized mRNA expression. Error bars indicate standard error. Data from *n* = 3 independent experiments, each performed in triplicate with independent donors of BC. **e** qPCR analysis to assess mRNA expression of BC markers (KRT5, TP63), secretory cell markers (MUC5AC, MUC5B, SCGB1A1), ciliated cell markers (FOXJ1, DNAI1) and squamous cell markers (KRT6B, IVL) at ALI days 7, 14, and 28. Data from *n* = 3 independent experiments, each performed in triplicate with independent donors of BC. Bars indicate the normalized mRNA expression. Error bars indicate standard error of the mean. Asterisks indicate *p* < 0.05 (*) or *p* < 0.001 (**). ANOVA was used to determine the statistical significance among groups as described in the methods section

To assess the role of increased KRAS expression or constitutive KRAS signaling on regulating BC differentiation, lentivirus vectors were utilized to overexpress wild-type (WT) KRAS or the constitutively active G12 V mutant (activated) during ALI culture. As a control, BC were infected with control lentivirus (empty vector). To confirm over-expression of WT and activated KRAS throughout the differentiation process, cells were harvested at ALI day 0, 7, 14 and 28 for analysis of KRAS expression at the mRNA level by qPCR. Compared to lentivirus control infected cells, KRAS was significantly over-expressed in WT and activated lentivirus infected cells at all time points (*p* < 0.05, Fig. 4a). No significant difference in KRAS expression was observed between WT and activated lentivirus infected cells (Fig. 4a).

**Fig. 5:**
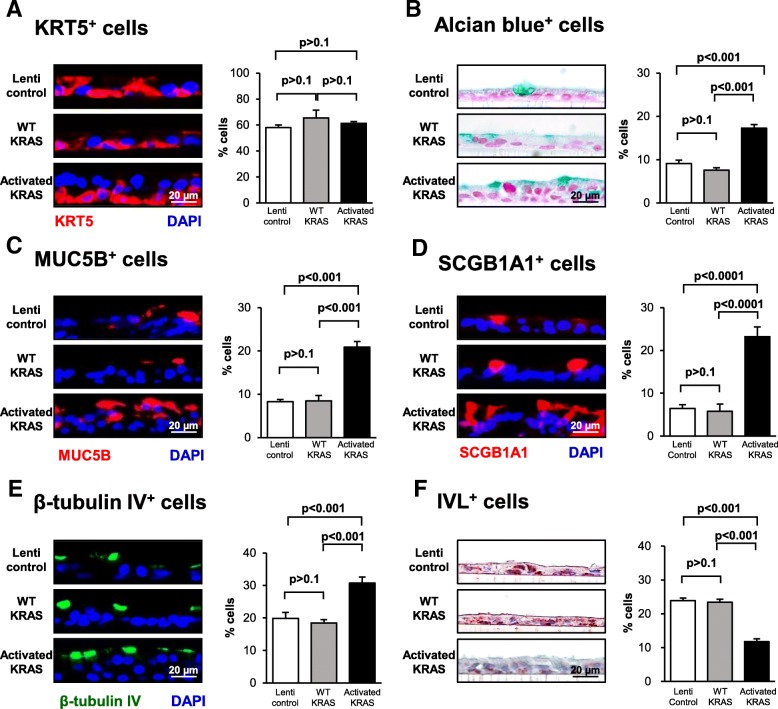
Consequences of constitutive KRAS activity on promotion of basal cell (BC) differentiation into secretory and ciliated cells Primary human airway BC were infected with control lentivirus or lentivirus over-expressing wild-type (WT) KRAS or the constitutively active G12 V mutant (activated) and cultured on ALI for 28 days to assess the impact of KRAS on BC differentiation into a mucociliated epithelium. **a** Immunofluorescence staining of KRT5^+^ BC. Sections of cells on ALI day 28 membranes were stained for KRT5 (red) and DAPI (nuclei, blue). **b** Alcian blue staining of secretory cells. Sections of cells on ALI day 28 membranes were stained for Alcian blue (blue). **c** Immunofluorescence staining of MUC5B^+^ secretory cells. Sections of cells on ALI day 28 membranes were stained for MUC5B (red) and DAPI (nuclei, blue). **d** Immunofluorescence staining of SCGB1A1^+^ secretory cells. Sections of cells on ALI day 28 membranes were stained for SCGB1A1 (red) and DAPI (nuclei, blue). **e** Immunofluorescence staining of β-tubulin IV^+^ ciliated cells. Sections of cells on ALI day 28 membranes were stained for β-tubulin IV (green) and DAPI (nuclei, blue). **f** Immunohistochemical staining of IVL^+^ squamous cells. Sections of cells on ALI day 28 membranes were stained for IVL (red) and DAPI (nuclei, blue). The data for **a**-**f** are the mean for *n* = 3 independent experiments performed with independent donors of BC. The bars indicate the mean percentage of positively stained cells for *n* = 3 independent experiments, and error bars indicate standard error of the mean. ANOVA was used for statistical comparisons among groups as described in the Methods. Scale bar 20 μm

To assess the effect of over-expressing WT and activated KRAS on BC differentiation at the histological level, ALI day 28 cross-sections were harvested and stained with H&E. The cells infected with control lentivirus generated a normal multi-layered mucociliated epithelium (Fig. 4b) and over-expression of WT KRAS resulted in the development of a mucociliated epithelium with no significant difference in thickness compared to lentivirus control infected cells (*p* > 0.1, Fig. 4c). In contrast, over-expression of activated KRAS during the differentiation process resulted in a significantly thicker mucociliated epithelium compared to control and WT KRAS expressing cells (both 2.5-fold, *p* < 0.001, Fig. 4c). In support of these findings, qPCR analysis of the proliferation marker MKI67 demonstrated no significant (*p* > 0.8) difference in expression between control lentivirus and lentivirus expressing WT KRAS infected cells. However, over-expression of activated KRAS increased expression of MKI67 compared to control (3.3 fold) and WT KRAS (3.3 fold) lentivirus infected cells suggesting activation of KRAS increases proliferation (Fig. 4d). However, despite a clear trend in expression differences between conditions the results were not significant (*p* > 0.17). To further characterize these differences in histology and quantify the impact of KRAS signaling on BC mucociliated differentiation, ALI day 7, 14, and 28 cultures from each group were analyzed by qPCR for expression of cell type specific markers. In support of the histological data, no significant difference in expression of BC (KRT5 and TP63), secretory (MUC5AC, MUC5B and SCGB1A1), ciliated (FOXJ1 and DNAI1) and squamous (KRT6B and IVL) cell markers was observed between cells infected with control lentivirus and lentivirus expressing WT KRAS at any time point (Fig. 4e). Compared to control lentivirus infected cells, over-expression of activated KRAS had no significant effect on expression of the BC markers KRT5 and TP63 at any time point (Fig. 4e; all *p* > 0.05). However, activated KRAS significantly increased expression of secretory cell markers at all time points; at day 28, the magnitude of increase for key genes included 7.4-fold for MUC5AC, 6.0-fold for MUC5B, and 2.9-fold for SCGB1A1(all *p* < 0.05). In ciliated cells, differentiation-driving transcription factor FOXJ1 was up-regulated 1.7-fold at day 7 (*p* < 0.001), and in parallel, cilia structural gene DNAI1 showed significant upregulation of 3.7- and 2.0-fold at days 7 and 14. By day 28, ciliated cell marker gene expression was higher than WT KRAS and control samples, but the difference was no longer significant. At the same time, squamous cell markers were downregulated in cultures expressing activated KRAS at day 28 compared with cultures expressing WT KRAS or control lentivirus infected cells (− 1.8-fold KRT6B and − 4.2-fold IVL). Differentiation changes observed at the mRNA level were subsequently validated histologically by staining of ALI sections at day 28. Immunofluorescent staining of the BC marker KRT5 demonstrated no significant difference (all *p* > 0.1) in positive cells between control, WT and activated KRAS over-expressing cells (58.0% lenti control vs 65.5% WT KRAS vs 61.4% in activated KRAS-expressing cultures; Fig. 5a). Significant increases (all *p* < 0.001) were observed in the number of Alcian blue positive (9.1% lenti control vs 7.6% WT KRAS vs 17.3% in activated KRAS-expressing cultures), MUC5B positive (8.3% lenti control vs 8.5% WT KRAS vs 20.9% in activated KRAS-expressing cultures) and SCGB1A1 positive secretory cells (6.5% lenti control vs 5.8% WT KRAS vs 23.2% in activated KRAS-expressing cultures; Fig. 5b-d). The proportions of β-tubulin IV positive ciliated cells were also enhanced (30.7%) relative to their numbers in lenti-control cells (19.9%) and lenti-WT KRAS-expressing cultures (18.4%) (Fig. 5e). Conversely, significant decreases (all p < 0.001) in the number of IVL positive squamous cells was observed (23.9% lenti control vs 23.4% WT KRAS vs 11.8% in activated KRAS-expressing cultures) (Fig. 5f).

Overall, these data suggest that over-expression of WT KRAS has no effect on BC differentiation into a mucociliated epithelium. However, over-expression of activated KRAS increases differentiation into secretory and ciliated cells with a corresponding decrease in squamous cell differentiation.

### Over-expression of activated KRAS has no effect on 518 expression of SOX family transcription factors and NOTCH 519 pathway genes

KRAS signaling regulates multiple cellular processes in the human and murine lung via modulating expression of the SOX family transcription factors (SOX2 and SOX9) [38–43] and interaction with additional signaling pathways including NOTCH [38]. To assess the involvement of SOX2, SOX9 and the NOTCH pathway on KRAS dependent regulation of BC differentiation we assessed their expression in response to over-expression of activated KRAS. Compared to control and WT KRAS lentivirus infected cells, over-expression of activated KRAS had no significant effect on expression of SOX2, SOX9 or the NOTCH pathway genes NOTCH1, 2, 3, HES1, HEY1 and HEY2 (Additional file 1: Figure S1; all *p* > 0.5). These data suggest that KRAS-dependent regulation of normal BC differentiation into a mucociliated epithelium involves downstream signaling mechanisms independent of SOX2, SOX9 and the NOTCH signaling pathway.

### Cigarette smoke exposure increases activation of KRAS and the RAS protein family in the airway epithelium in vitro and in vivo

**Fig. 6:**
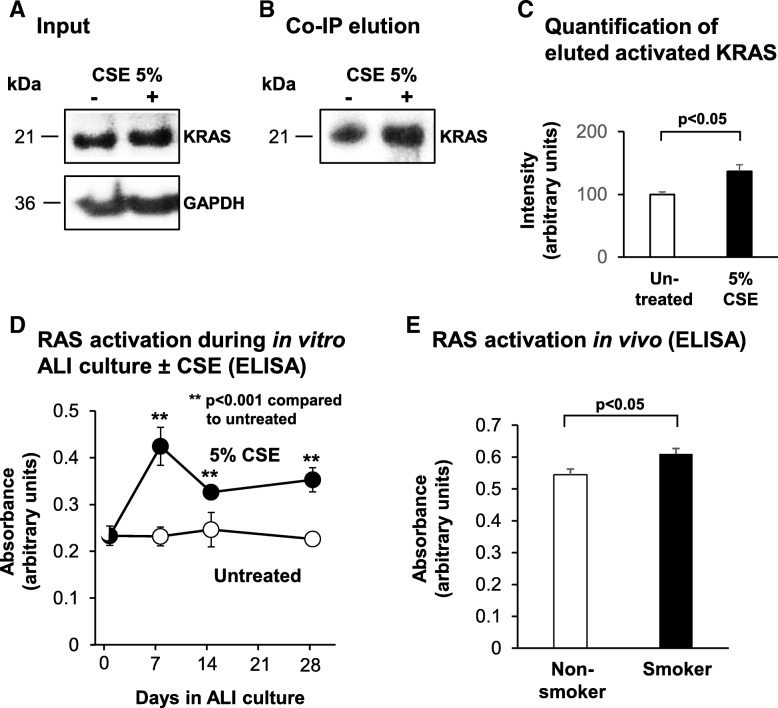
Effect of cigarette smoke exposure on KRAS and RAS protein family activation in the airway epithelium. **a**-**c**. Basal cells (BC) were cultured in the absence or presence of 5% cigarette smoke extract (CSE) under non-differentiating culture conditions. Forty-eight hr. post-CSE exposure, the cells were lysed and the activated form of GTP-bound KRAS quantified by co-immunoprecipitation (Co-IP). **a** Western analysis of KRAS and GAPDH (loading control) levels in cell lysates. **b** Western analysis of activated KRAS levels elution lysates following Co-IP. **c** Quantification of activated KRAS levels. Bars indicate intensity of signal obtained in during image analysis. Error bars indicate standard error of the mean. Data from *n* = 3 independent experiments, each performed with independent donors of BC. **d** Basal cells were cultured on ALI for 28 days in the absence and presence of 5% CSE and harvested at multiple time points to quantify RAS protein family activation by ELISA. Bars indicate mean absorbance levels. Error bars indicate standard error of the mean. Data from *n* = 3 independent experiments, each performed in triplicate with independent donors of BC. **e** Airway epithelium from healthy nonsmokers (*n* = 5) and asymptomatic healthy smokers (*n* = 5) were obtained via bronchoscopic brushing and the levels of RAS protein family activation quantified by ELISA. Bars indicate mean absorbance levels. Error bars indicate standard error of the mean. T tests were used to determine the statistical significance of differences observed in two-way comparisons. Statistical analysis of data in the time course utilized a repeated measures ANOVA as described in the Methods

To assess the effect of cigarette smoke exposure on KRAS activation in the airway epithelium in vitro, BC were cultured in the absence or presence of 5% cigarette smoke extract (CSE) under non-differentiating culture conditions. Forty-eight hours post CSE exposure, the cells were lysed and the activated form of GTP-bound KRAS quantified by co-immunoprecipitation (co-IP) and subsequent elution using the GST-Raf1-RBD (Rho binding domain) fusion protein and glutathione resin. Western analysis for the input cell lysates used for the Co-IP showed equal amounts of total KRAS protein in untreated and CSE treated cells (Fig. 6a). However, Western analysis of the co-IP elutions demonstrated increased levels of activated KRAS in the CSE treated cells (Fig. 6b). Quantification of these levels demonstrate that compared to untreated cells, CSE exposure significantly increased KRAS activation (36.7%, *p* < 0.001, Fig. 6c). To determine the effect of cigarette smoke exposure on KRAS activity during BC differentiation, BC were cultured on ALI for 28 days in the absence and presence of 5% CSE and harvested at multiple time points. Due to the large amounts of cell lysate required to quantify activated KRAS levels by co-IP, we were unable to use this assay for samples cultured on ALI. Instead, we used a more sensitive ELISA method that quantifies activation of all RAS family proteins (KRAS, HRAS and NRAS). The results demonstrated that compared to untreated cells, CSE exposure significantly (all *p* < 0.001) increased levels of activated RAS at ALI day 7 (83% increase), day 14 (32% increase) and day 28 (56% increase; Fig. 6d). Using the same ELISA assay, we next assessed the effect of cigarette smoke exposure on RAS protein family activation in the airway epithelium in vivo. Analysis of airway epithelium brushings isolated via bronchoscopy from the large airway of nonsmokers and asymptomatic healthy smokers demonstrated a significant increase in RAS activation (12% increase, *p* < 0.05) in the airway epithelium of smokers compared to nonsmokers (Fig. 6e). Combined, these data demonstrate that cigarette smoke exposure increases KRAS and RAS protein family activation in the airway epithelium in vitro and in vivo*.*

## Discussion

KRAS is a member of the RAS protein family, a class of small GTP-binding proteins with intrinsic GTPase activity that function as molecular switches for multiple cellular processes [1–5]. Cancer-associated driver mutations in KRAS lock the protein into the GTP-bound state preventing the hydrolysis of GTP to GDP, resulting in constitutive signaling which give mutated cells a growth advantage and leads to development of cancer [5–8]. Despite the extensive knowledge on the role of KRAS mutations in the pathogenesis of lung cancer [8–11], little is known about the role of KRAS-dependent signaling in regulating normal human airway epithelial function or the impact of cigarette smoke exposure on KRAS activation. The present study was designed to understand the role of KRAS-dependent signaling on regulation of BC differentiation into a mucociliated epithelium and determine the effect of cigarette smoke exposure on KRAS activation in the human airway epithelium both in vitro and in vivo*.*

### KRAS-dependent regulation of BC differentiation

To assess the role of KRAS signaling in regulating differentiation of the human airway epithelium, BC were cultured on ALI and KRAS signaling was either suppressed during differentiation via siRNA-mediated knockdown of KRAS expression or activated via over-expression of the constitutively active G12 V KRAS mutant. Suppression of KRAS expression resulted in production of a leaky epithelium that failed to regenerate a fully differentiated mucocilated epithelium and survive for 28 days. However, analysis of differentiation following 14 days of culture when the cells were still viable demonstrated a thinner epithelium following KRAS knockdown with decreased numbers of non-mucus producing secretory (SCGB1A1^+^) cells and complete loss of mucus-producing secretory (Alcian blue^+^ and MUC5B^+^) cells and ciliated (β-tubulin IV^+^) cells with a corresponding increase in squamous (IVL^+^) cells. Conversely, constitutive KRAS activation via over-expression of the G12 V KRAS mutant had the opposite effect and produced a thicker epithelium with increased numbers of secretory (Alcian blue^+^, MUC5B^+^ and SCGB1A1^+^) and ciliated (β-tubulin IV^+^) cells with a decreased number of squamous (IVL^+^) cells. Interestingly, over-expression of wild-type KRAS had no effect on BC differentiation suggesting feedback mechanisms exist to tightly control KRAS signaling in the presence of elevated protein levels. Overall, these data demonstrate that KRAS signaling is critical for regulating differentiation of BC into a mucociliated epithelium, with suppression of KRAS signaling increasing squamous cell differentiation at the expense of secretory and ciliated cell differentiation. In contrast, activation of KRAS signaling promotes secretory and ciliated cell differentiation at the expense of squamous cells.

KRAS-dependent signaling involves multiple downstream signaling pathways including those mediated by RAF/MEK/ERK and PI3K/AKT to regulate multiple cellular functions [2, 12–15]. Studies in the developing mouse demonstrate that KRAS-dependent signaling plays a critical role in regulating branching morphogenesis and proximal-peripheral cell fate decisions [39, 40]. Expression of constitutively activated Kras^G12D^ in the respiratory epithelial impaired branching morphogenesis via increased activation of ERK signaling which phosphorylates Foxm1 to induce its transcriptional activity to regulate these processes [40, 41]. Activation of the Kras/ERK/Foxm1 signaling pathway inhibits canonical Wnt/β-catenin signaling which plays a critical role in regulating the position of epithelial precursors along the proximal (SOX2^+^) and peripheral (SOX9^+^) axis of the lung [20, 40, 42, 43]. Suppression of Wnt/β-catenin signaling via Kras/ERK/Foxm1 activation results in accumulation of atypical SOX9^+^ BC in the proximal airways and subsequent disruption of normal morphogenesis [43]. Furthermore, in the context of lung cancer the subtype of KRAS-induced tumors (i.e., adenocarcinoma vs squamous carcinoma) is regulated by activation of the NOTCH signaling pathway and expression of SOX2 [38]. However, based on our data demonstrating that over-expression of activated KRAS in differentiating BC has no effect on expression of SOX2, SOX9 and multiple NOTCH pathway genes, we hypothesize that KRAS regulates differentiation of normal human adult BC into secretory and ciliated cells independent of FOXM1/WNT, SOX2/SOX9 and NOTCH signaling.

### Cigarette smoking induces KRAS activation

Based on the knowledge that KRAS activation plays a critical role in regulating BC differentiation into a mucociliated epithelium and that cigarette smoking is a major driver of airway epithelial remodeling, we assessed the effect of cigarette smoke exposure on KRAS activation. Short term exposure of BC to cigarette smoke extract (CSE) in vitro resulted in increased KRAS activity compared to untreated cells. Furthermore, CSE treatment of BC during differentiation on ALI culture had the same effect and increased activation of the RAS protein family. These in vitro findings were then validated in vivo using airway epithelial brushings isolated via bronchoscopy from the airway of nonsmokers and asymptomatic healthy smokers, which demonstrated increased RAS activation in the airway epithelium of smokers. Based on these observations, we conclude that cigarette smoke exposure increases KRAS (and RAS protein family) activation in the human airway epithelium. Upon exposure to cigarette smoke, the airway epithelium becomes progressively disordered which impairs its structure and function [23, 44–49]. One hallmark of these changes includes increased numbers of mucus-producing secretory cells defined as mucus or goblet cell hyperplasia [44–49]. Based on our data that KRAS activation in BC promotes increased differentiation of mucus-producing secretory cells and that cigarette smoking increases levels of activated KRAS, we hypothesize that cigarette-smoke induced goblet cell hyperplasia is driven in part by KRAS signaling dependent mechanisms.

In addition to contributing to the process of airway epithelial remodeling, cigarette smoke induced activation of KRAS may play an important role in oncogenic transformation of the airway. A recent study by Vaz et al. [50] using untransformed human airway epithelial cells demonstrated that in vitro long term exposure of the cells to cigarette smoke condensate induces epigenetic changes commonly seen in smoking-related non-small cell lung cancer which sensitize the cells to transformation with a single KRAS driver mutation. Based on our findings, we can hypothesize that cigarette smoke-induced activation of wild-type KRAS may contribute to the sensitization of the cells to oncogenic transformation via activation of growth promoting pathways.

## Conclusion

In summary, our data demonstrates that KRAS-dependent signaling plays a critical role in regulating the balance of secretory, ciliated and squamous cell differentiation of the human airway epithelium. Suppression of KRAS signaling increased squamous cell differentiation at the expense of secretory and ciliated cell differentiation, whereas activation of KRAS signaling promotes secretory and ciliated cell differentiation at the expense of squamous cells. Furthermore, cigarette smoke exposure results in increased KRAS activity in the airway both in vitro and in vivo. Therefore, development of airway epithelial remodeling in smokers may, in part, be regulated by cigarette smoke-mediated activation of KRAS-dependent signaling in BC.

## Additional file

Additional file 1: **Figure S1.** Effect of constitutive KRAS activity on expression of SOX family tran-scription factors and NOTCH pathway genes. (PDF 239 kb)

ᅟ

## Data Availability

Please contact the corresponding author.
