# Supplementary material for: Role of KRAS in regulating normal human airway basal cell differentiation
Source: Respir Res. 2019 Aug 9;20:181. doi: 10.1186/s12931-019-1129-4 (PMC6688249; doi:10.1186/s12931-019-1129-4)

Supplemental Methods

Inclusion and Exclusion Criteria

Healthy controls: Inclusion criteria

- Males and females, at least 18 years old
- Provide informed consent
- Good health without history of chronic lung disease, including asthma, and without recurrent or recent (within 3 months) acute pulmonary disease
- Normal physical examination
- HIV negative
- Normal α1-antitrypsin level
- Not pregnant (females)
- Never-smokers by history, with current smoking status validated by the following metabolites findings: urine nicotine <2 ng/ml and urine cotinine <5 ng/ml
- Normal routine laboratory evaluation, including general hematologic studies, general serologic/immunologic studies, general biochemical analyses and urine analysis
- Forced expiratory volume in 1 second (FEV1) ≥80% predicted, forced vital capacity (FVC) ≥80% predicted, FEV1/FVC (≥0.7) based on pre-bronchodilator spirometry and total lung capacity (TLC) ≥90% predicted
- Normal posterior-anterior (PA) and lateral chest radiograph
- Normal electrocardiogram
- No history of allergies to medications used during bronchoscopy
- Not taking any medications relevant to lung disease or known to effect the airway epithelium
- Willingness to participate in the study

Smokers: Inclusion criteria

- Males and females, at least 18 years old
- Provide informed consent
- Normal physical examination
- HIV negative
- Normal α1-antitrypsin level
- Not pregnant (females)
- Self-reported current daily smokers with any number of pack-years, validated by urine nicotine ≥30 ng/ml or cotinine ≥50 ng/ml
- Forced expiratory volume in 1 second (FEV1) ≥80% predicted, forced vital capacity (FVC) ≥80% predicted, FEV1/FVC (≥0.7) based on pre-bronchodilator spirometry and total lung capacity (TLC) ≥80% predicted
- Normal PA and lateral chest radiograph
- Normal electrocardiogram
- Good health without history of chronic lung disease, and without recurrent or recent (within 3 months) acute pulmonary disease
- No history of allergies to medications used during bronchoscopy
- Normal routine laboratory evaluation, including general hematologic studies, general serologic/immunologic studies, general biochemical analyses, and urine analysis
- Willingness to participate in the study

All subjects: Exclusion criteria

- Unable to meet the inclusion criteria
- Individuals in whom participation in the study would compromise the normal care and expected progression of their disease
- Current active infection or acute illness of any kind
- Evidence of malignancy within the past 5 years other than localized skin malignancy
- Current alcohol or drug abuse
- Any evidence of interstitial lung disease, pulmonary hypertension, diastolic dysfunction or other disorders associated with a low DLCO

Supplemental Figure Legend

Supplemental Figure 1. Effect of constitutive KRAS activity on expression of SOX family transcription factors and NOTCH pathway genes. Primary human airway BC were infected with control lentivirus or lentivirus over-expressing wild-type (WT) KRAS or the constitutively active G12V mutant (activated) and cultured on ALI for 14 days **A.** qPCR analysis to assess mRNA expression of SOX2 and SOX9. **B.** qPCR analysis to assess mRNA expression of the NOTCH pathway genes NOTCH1, NOTCH2, NOTCH3, HES1 and HEY1. For **A** and **B** bars indicate the normalized mRNA expression. Error bars indicate standard error. Data from n=3 independent experiments, each performed in triplicate with independent donors of BC.

A. mRNA expression of SOX transcription factors

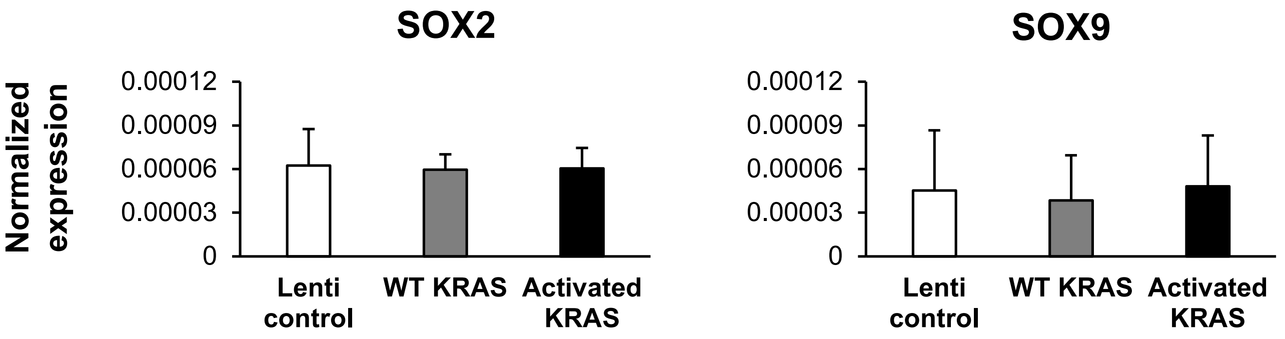

B. mRNA expression of NOTCH pathway genes

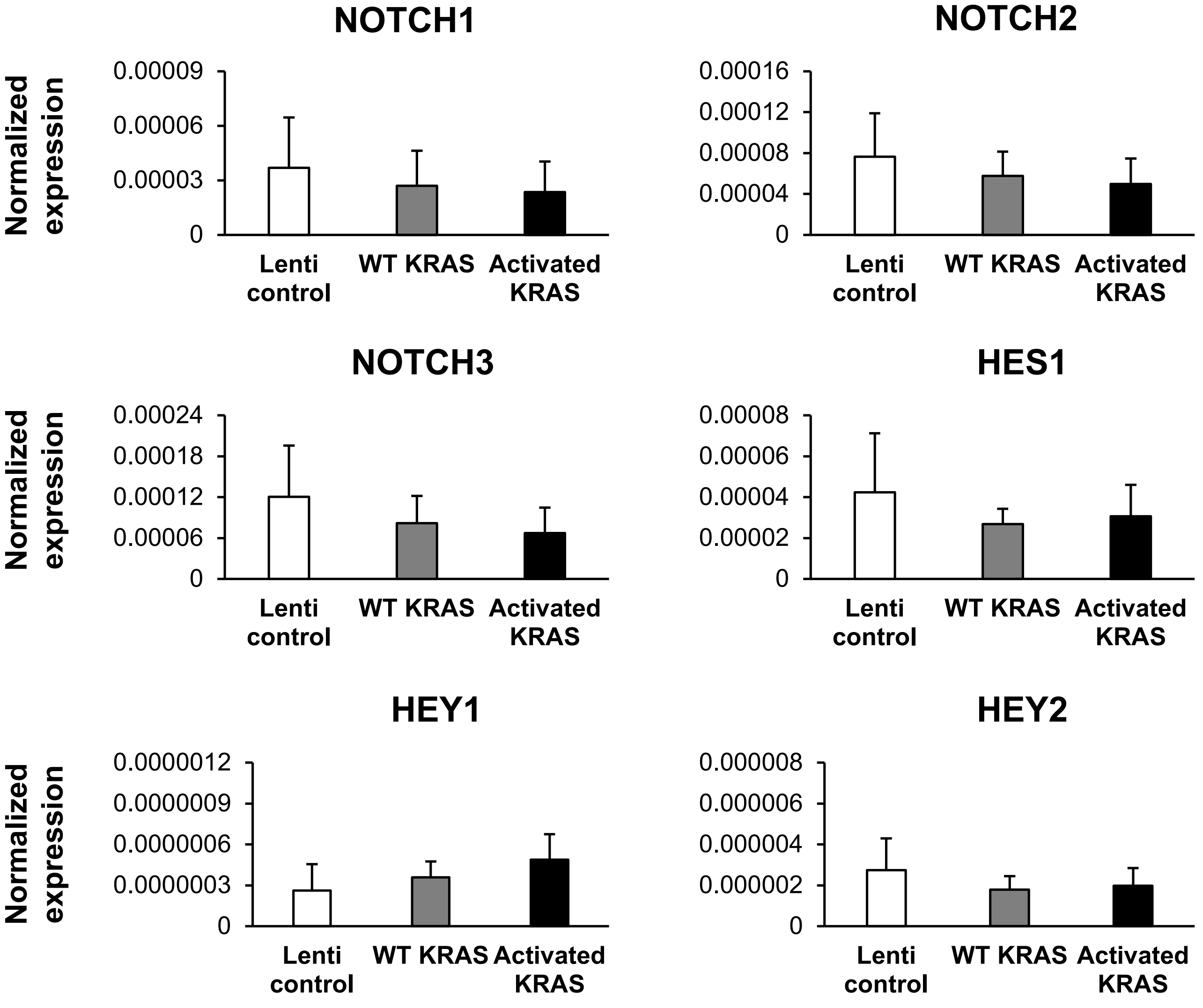

Supplement: Supplementary file 1 — Figure S1. Effect of constitutive KRAS activity on expression of SOX family tran-scription factors and NOTCH pathway genes. (PDF 239 kb) [file 12931_2019_1129_MOESM1_ESM.pdf]
